# Analgesic and Antibiotic Prescription Pattern among Dentists in Guangzhou: A Cross-Sectional Study

**DOI:** 10.1155/2020/6636575

**Published:** 2020-12-29

**Authors:** Jiali Yu, Er-Min Nie, Rui Jiang, Chun-Yuan Zhang, Xiang Li

**Affiliations:** First Affiliated Hospital, Sun Yat-Sen University, 58 Zhong Shan Road 2nd, Guangzhou 510080, China

## Abstract

**Aim:**

To assess the rational use of drugs and the pattern of prescribing of analgesics and antibiotics for dental management and the information given by dentists in Guangzhou to their patients about the use of these drugs.

**Methods:**

A questionnaire was distributed to 225 dentists working in Guangzhou. The questionnaires consisted of open-ended questions and were given to dentists about analgesic and antibiotic use in dentistry. The questionnaires were analyzed, and absolute frequencies were expressed in the answers to each question. The cases, the analgesics, and the antibiotics recommended by the dentists for each case were determined by the frequency analysis method of descriptive statistics.

**Results:**

Responses to the questionnaire were received from 164 (72.9%) dentists. Paracetamol and diclofenac were the most widely prescribed analgesics. It is also estimated that selective COX-2 inhibitors or opioid analgesics have not been administered by dentists. The antibiotics primarily used for treatment were amoxicillin and metronidazole, and amoxicillin was used for prophylaxis. While more than 80% of dentists indicated that they provided their patients with information on the use of antibiotics, the quality of the information was limited. Patients were primarily instructed by dentists to observe the dosage and dose intervals of the prescription drugs.

**Conclusions:**

The results of the present study demonstrated that dentists most commonly prescribe paracetamol and diclofenac as analgesics, amoxicillin, and metronidazole for the therapy of periodontal, endodontic, and surgical procedures. The results also showed that dentists informed their patients inadequately about analgesic and antibiotic use.

## 1. Introduction

Pain is a main reason for which dental care is sought by patients. Pain may be arisen from different structural or anatomical origins of odontogenic or nonodontogenic sources. Most cases are related to the treatment of the pulpal pathology. Diagnosing and eradicating the cause is the main task for a dental surgeon. Usually, pain management is followed by the 3 “D” principle of diagnosis, dental treatment, and drugs [1]. The dentist often prescribes analgesics and antibiotics to patients for multiple causes, which can be a surgical or non-surgical purpose [2].

Nonopioid analgesics: paracetamol is widely used in dental pain as an antipyretic analgesic along with nonsteroidal anti-inflammatory medications (NSAIDs). In the treatment of mild to extreme dental pain, opioid analgesics are often rarely used [3, 4]. NSAIDs are very helpful for initial inflammatory pain and offer outstanding analgesia for mild to moderate pain. These medications are widely used for dental pain due to their analgesic and anti-inflammatory effects [5]. The effects of NSAIDs are inhibited by cyclooxygenase (COX) enzymes that have a primary role in prostaglandin and other eicozanoid syntheses. In a recent publication, NSAIDs (both selective COX-2 and nonselective COX inhibitors) were advised to be used with caution in heart disease patients. The widespread use of these medications in the treatment of dental pain raises the risk of adverse reactions related to these drugs (including bronchopulmonary, gastrointestinal, renal, and hematological) [6].

In dentistry, prescribing antibiotics is typically empirical, i.e., because culture tests are not widely conducted, the clinician does not identify the responsible organism. As a result, broad-spectrum antibiotics are widely used, and the emergence of the antibiotic resistance for the oral microorganism has increased [4]. In addition to the development of resistance, other issues with the use of antibiotics include adverse reactions (including gastrointestinal, allergic, and hematologic reactions). For this purpose, the rational use of antibiotics in oral or dental practice is important for reducing the growth of resistance in oral pathogens and the risk of adverse effects while increasing efficacy. Informing patients properly about the prescription medications is another increasing effectiveness parameter. This information concerns not just the dosage and treatment times but also the adverse effects, medication interactions, conditions of storage, and the cost of the prescription medications. It also involved briefing of these explanations by patients when dentists told their patients about these subjects [7]. This prevents the information provided from being confused. On the contrary, perfect knowledge would improve the patient treatment's quality, patient compliance, quality of life, and cost-effectiveness [8, 9].

There is an irregularity among practitioners regarding the length, drug choice, frequency, and necessity of prescribing them based on the literature review [10]. Since a significant amount of dental pain originates from acute or chronic pulpal infections, a significant amount of information needs operative intervention and should be known with analgesics and antibiotics [11]. The need for analgesics and antibiotics is confusing for many practitioners [2]. Antibiotic therapy regulates infection pathways to establish, avoid, or create favourable conditions for species to remove bacterial and fungal contingents through their immunological mechanisms [12]. There is no standard universal prescribing regimen for antibiotics and analgesics before, during, and after dental treatment. Therefore, the purpose of this study was to evaluate the pattern of analgesics and antibiotics by a practicing dentist in and around the population of Guangzhou under different conditions.

## 2. Materials and Methods

### 2.1. Questionnaire

To analyze the analgesic and antibiotic prescribing practices of dentists, a structured questionnaire was developed. As the degree of dental antibiotic and analgesic prescribing is unknown in Guangzhou, by using standardized proportion for the most conservative estimate of the sample size and with a 0.05 standard error and 95% confidence interval, 224 responses were required for this study. Dentists who are fully engaged with academic and nonclinical work or retired from their services were excluded from this study. The questionnaire was unidentifiable for any dental surgeon's identity. The questionnaire comprised four sections. Section A: demographic and characteristic information (age, gender, years of experience, postgraduation level, and working place information), Section B: most common analgesic-prescribing pattern (common analgesic available in generic name and clinical condition), Section C: most common antibiotic-prescribing pattern (common antibiotic available in generic name and clinical condition), and Section D: the information given to patients about the use of these drugs.

### 2.2. Sample and Data Handling

This study was approved by the Ethics Committee of the First Affiliated Hospital, Sun Yat-sen University, and the list of the dentists was received from the local dental council. To produce a homogeneous distribution, dentists in the list were chosen from different regions of Guangzhou. The questionnaires were immediately emailed to the dentists after their consent for participation. In June 2020, the distribution of the questionnaires began via email, and the delivery and selection processes ended in July 2020.

### 2.3. Statistical Analysis

Data from questionnaires received were entered into Statistical Package for Social Sciences® (SPSS), version 25.0. From this database, the overall response rate was calculated, together with the percentage responses for each question. Frequency analysis was used for the determination of the demographic, analgesic, and antibiotic prescribing pattern. Multiple linear regression analysis was performed to find out the association between the demographic and prescribing pattern.

## 3. Results

### 3.1. Demographics and Characteristics

A total of 164 replies (out of 225 questionnaires sent) were received giving a response rate of 72.9%. Out of the 164 respondents, 89 (54.3%) were males and 75 (45.7%) were females. Demographic and professional characteristics of respondents are shown in Table 1. The number of respondents who had attended any postgraduate education is 88 (53.7%) with 6–10 years of experience (32.3%). And as seen in Table 1, a majority of the respondents work at dental practices (98 (59.7%)).

### 3.2. Analgesic Prescribing Pattern

Table 2 shows the prescribing pattern of analgesics by the dentist. Among the participants, 54.9% of participants prescribed paracetamol analgesics for acute pulpitis, 45.7% of them prescribed diclofenac, and 25.6% of them prescribed naproxen analgesics in the acute apical abscess. 42.7% of dentists prescribed paracetamol, and 31.3% of dentists prescribed diclofenac analgesics in chronic pulpitis and 45.7% in chronic apical periodontitis with a sinus tract. 79% prescribed analgesics, mainly diclofenac and paracetamol, combined with caffeine (16.5%) in diffuse swelling. Diclofenac (18.3%) commonly prescribed prior root canal treatment.

### 3.3. Antibiotic Prescribing Pattern

Table 3 shows that the dentist prescribed amoxicillin for acute pulpitis (25%), acute apical abscess (80.1%), chronic pulpitis (19.5%), chronic apical periodontitis with the sinus tract (28.0%), diffuse swelling (78%), and 21.3% before root canal treatment. Along with amoxicillin, metronidazole was the second most commonly prescribed antibiotic by the dentist. 89.6% of dentists prefer metronidazole in diffuse swelling following acute apical abscess treatment (76.0%).

### 3.4. Information Given to the Patients

**Figure 1:**
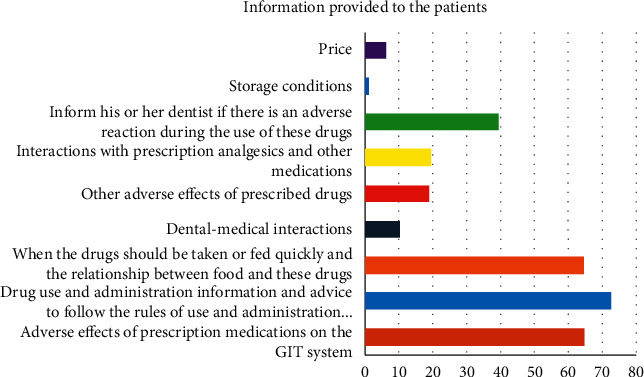
Information provided to the patients.

The majority of the respondents (80.1%) reported they gave information to their patients about analgesic and antibiotic use.  Table 4 shows the information given by respondents to their patients about analgesic and antibiotic use. As seen in the table, the most common information given by the respondents to their patients was “to obey the dose and dose interval rules given” (72.6%), “warning about the adverse reactions of these drugs on the gastrointestinal system” (64.7%), and “whether the drugs should be taken before or after the meal and the interactions between food and these drugs” (64.6%) (Figure 1).

### 3.5. Factors Associated with the Prescribing Pattern

Table 5 shows that demographic factors such as year experience and postgraduate education have a significant association with prescribing patterns. An experienced dentist without any postgraduate courses often prescribed inappropriate antibiotics and analgesics compared to the new trained and postgraduated dentist (*P*=0.001 − 0.004).

## 4. Discussion

Prescribing of antibiotics and analgesics by endodontists was assessed in this questionnaire-based cross-sectional study. The questions and endodontic conditions suggested in the questionnaire are like those in India [7], Turkey [13], and Spain [14]. The study showed a response rate of 72.9 percent, which is considered appropriate for questionnaire-based study.

Different studies have shown that NSAIDs are effective in lowering the dental pain threshold at different doses after, before, or just before root canal treatment [15, 16]. Thus, it is not surprising that pain relief analgesics were recommended by dentists in our research. Paracetamol and diclofenac were the most frequently prescribed analgesics listed in the questionnaire for various dental conditions.

An acetic acid derivative of diclofenac offers excellent analgesia for dental pain and is consistently reported in several studies [17]. Study results indicate that respondents do not have a prescribing pattern that involves selective inhibitors of COX-2. Without the undesirable side effects, COX-2 inhibitors induce desired anti-inflammatory effects, particularly gastric irritation associated with COX1 inhibitors, but clinical use of these drugs has resulted in increased cardiovascular risk [18, 19].

One of the most widely prescribed analgesics by dentists is paracetamol, which has a low risk of GIT bleeding and has even been shown to have the least anti-inflammatory effects on peripheral tissues. In this research, paracetamol leads to much of the endodontists' analgesics alongside diclofenac. In the report, respondents did not recommend opioid analgesics for pain. Opioid analgesics are used because of their detrimental effects and abuse in cases of extreme pain rather than in moderate pain [15].

In the above research, analgesics were significantly prescribed in cases of acute pulpitis, acute apical abscess, followed by chronic apical abscess with the sinus tract that may lead to the timely release of pain, while analgesics do not help to reduce the inflammatory process supported by the literature that treatment may improve pain relief without medication. It is also an important treatment technique [20] for the management of these conditions. It is widely agreed that antibiotics are not indicated if infection, systemic involvement, or immune-compromised disease is not present [21, 22].

In dental cases such as acute pulpitis, diffuse swelling, acute apical abscess, and retreat events, amoxicillin was widely administered in the sample. Amoxicillin is a moderate spectrum, bacteriolytic, *ß*-lactam antibiotic, which represents a molecule of synthetic penicillin. It is easily digested and can be swallowed with food. It is better able to avoid stomach acid damage so that less oral dose is lost. It has a much wider spectrum against the Gram-negative cell wall, and the cell wall will last longer [23]. It is the principal antibiotics dentist prescribed in the USA [24].

In the review of many dentists, metronidazole was the next antibiotic of choice, having an outstanding activity against anaerobes but no activity against aerobes. Metronidazole has shown the greatest bacterial resistance and is only effective against anaerobes, so it should not be prescribed alone for the treatment of endodontic infections [23]. The dosage and length of antibiotics recommended in the clinical recommendations are most often based on the expert opinion [7].

To avoid side effects of resistant strains, antibiotics should also be administered at the required dosage, dose, and length to achieve good minimum inhibitory concentrations. A common trend of prescription of antibiotics found in the present study was that, in cases of periapical involvement with the presence of essential pulp, there was an improper prescription of antibiotics that was not justified. In a survey conducted in Spain where 40 percent of respondents inappropriately administered antibiotics, similar findings were found [7]. The research showed a propensity towards overprescription and showed a lack of knowledge of the occurrence of adverse reactions among dentists [23].

The research also showed a change in prescribing antibiotics and analgesics with years of specialty practice. In contrast to endodontists with more than 10 years of experience, a dentist with an experience from 1 to 5 years overprescribed analgesics and antibiotics instead of clinical treatment modalities, which may be due to the degree of functional experience and awareness of the root cause of the condition, supported by a related study by Marra et al. [11]. The study found that analgesics and antibiotics were overly prescribed by the majority of dentists, whereas 1/4th of the dentists appropriately prescribed. There is greater concern about the indiscriminate use of analgesics and antibiotics. For a well-defined sign in dental infection, the use of them should be judicial [25]. To avoid abuse and overuse of analgesics and antibiotics, the dentist should have a sound understanding of endodontic conditions, so as not to add to the global issue of antibacterial resistance and to prevent the adverse effects of these medications [26].

## 5. Conclusion

The present study suggested that most dentists overprescribe analgesics and antibiotics, but few prescribe them properly. The dentist's overprescription of analgesics and antibiotics in Guangzhou may be due to the lack of scientific awareness of the condition and pharmacology of the medication, patient demand, or other unknown factors. The fact that overprescribing medicines in Guangzhou should therefore be of concern. This research collaborates with other studies that recognize prescription protocol problems [11].

## Figures and Tables

**Table 1:** Demographics and characteristics of respondents.

Variable	*N* (%)
Gender	
Male	89 (54.3)
Female	75 (45.7)

Age	
21–30	50 (30.5)
31–40	71 (43.3)
41–50	34 (20.7)
51–60	9 (5.5)

Years of experience	
0–5	46 (28.0)
6–10	53 (32.3)
11–20	44 (26.9)
21–30+	21 (12.8)

Postgraduation education	
Yes	88 (53.7)
No	76 (46.3)

Postgraduation level	
Postgraduate courses	40 (24.4)
Masters	36 (21.3)
Doctorate	12(7.3)

Place of works	
Private dental practice	98 (59.7)
Private institution	19 (11.6)
University affiliated hospital	36 (22.0)
Government facility	11 (6.7)

**Table 2:** Prescribing pattern of analgesics under different conditions.

Analgesic	Dental condition, *n* (%)					
	Acute pulpitis	Acute apical abscess	Chronic pulpitis	Chronic apical periodontitis with the sinus tract	Diffuse swelling	Prior to root canal treatment
Paracetamol	90 (54.9)	0 (0.0)	70 (42.7)	30 (18.3)	12 (7.3)	0 (0.0)
Naproxen	15 (9.1)	42 (25.6)	18 (11.0)	0 (0.0)	0 (0.0)	0 (0.0)
Diclofenac	35 (21.3)	75 (45.7)	51 (31.1)	20 (12.2)	60 (36.9)	30 (18.3)
Paracetamol-caffeine combination	10 (6.1)	40 (24.4)	7 (4.3)	20 (12.2)	27 (16.5)	0 (0.0)
Etodolac	9 (5.5)	7 (4.3)	15 (9.1)	5 (3.0)	30 (18.3)	0 (0.0)
Ketoprofen	5 (3.0)	0 (0.0)	3 (1.8)	0 (0.0)	0 (0.0)	0 (0.0)

**Table 3:** Prescribing pattern of antibiotics under different conditions.

Variable	Dental condition, *n* (%)					
	Acute pulpitis	Acute apical abscess	Chronic pulpitis	Chronic apical periodontitis with the sinus tract	Diffuse swelling	Prior to root canal treatment
Amoxicillin	41 (25.0)	131 (80.1)	32 (19.5)	46 (28.0)	128 (78)	35 (21.3)
Clindamycin	2 (0)	25 (15.3)	0 (0)	0 (0)	0 (0)	18 (29.5)
Metronidazole	0 (0)	125 (76.0)	6 (3.7)	72 (0)	147 (89.6)	0 (0)
Erythromycin	0 (0)	4 (2.4)	0 (0)	12 (7.3)	2 (1.2)	0 (0)

**Table 4:** Information given to the patients by the dentist about prescribed antibiotics and analgesics.

Information provided to the patients	*N* (%)
Adverse effects of prescription medications on the GIT system	106 (64.7)
Drug use and administration information and advice to follow the rules of use and administration provided	119 (72.6)
When the drugs should be taken or fed quickly and the relationship between food and these drugs	106 (64.6)
Dental-medical interactions	17 (10.4)
Other adverse effects of prescribed drugs	31 (19)
Interactions with prescription analgesics and other medications	32 (19.6)
Inform his or her dentist if there is an adverse reaction during the use of these drugs	65 (39.6)
Storage conditions	2 (1.3)
Price	10 (6.2)

**Table 5:** Demographic factors associated with the prescribing pattern under different clinical conditions.

Variable	Adjusted OR	95% confidence interval		*P* value
		Lower	Upper	
Acute pulpitis	2.79	1.16	4.54	0.004
Acute apical abscess	4.11	1.59	23.51	0.001
Acute apical abscess	2.53	1.02	6.25	0.006
Chronic apical periodontitis with the sinus tract	1.12	0.66	5.16	0.001
Diffuse swelling	0.33	0.18	0.61	0.001
Prior to root canal treatment	0.28	0.09	0.59	0.027

## Data Availability

The data used to support the findings of this study are available from the corresponding author upon request.
